# Managing Pediatric Acquired Hemophilia A When Guideline-Recommended Bypassing Agents Are Unavailable: A Case Report

**DOI:** 10.3390/jcm15207859

**Published:** 2026-10-12

**Authors:** Li Zhang, Xiao-Li Guo, Jian-Wen Xiao, Yu-Xia Guo

**Affiliations:** 1 Department of Hematology and Oncology, Children’s Hospital of Chongqing Medical University, National Clinical Research Center for Children and Adolescents’ Health and Diseases, Ministry of Education Key Laboratory of Child Development and Disorders, Chongqing 400014, China; 2 Intelligent Application of Big Data in Pediatrics Engineering Research Center of Chongqing Education Commission of China, Chongqing 400014, China

**Keywords:** acquired hemophilia A, child, factor VIII inhibitor, hemostatic management, case report

## Abstract

**Background/Objectives:** Acquired hemophilia A (AHA) is caused by neutralizing factor VIII (FVIII) autoantibodies and is exceptionally rare in children. Pediatric treatment evidence is limited, and recommended hemostatic agents may be inaccessible. **Case:** An 11-year-old previously healthy girl presented with soft-tissue, mucosal, and genitourinary bleeding, an isolated markedly prolonged activated partial thromboplastin time, FVIII activity of approximately 1.4–2%, and an FVIII inhibitor. Recombinant activated factor VII was financially inaccessible to the family, and activated prothrombin complex concentrate (APCC) was unavailable. After a multidisciplinary discussion with the family, non-activated four-factor prothrombin complex concentrate (4F-PCC) was administered off-label; corticosteroid-based treatment was initiated to eradicate the inhibitor. **Results:** Gross hematuria resolved during the treatment period, and microscopic hematuria cleared subsequently. By July 2025, FVIII activity was 125%, the inhibitor was undetectable, immunosuppression had been withdrawn, and no recurrent major AHA-related bleeding had been reported. **Conclusions:** This case illustrates individualized decision-making in pediatric AHA when recommended hemostatic agents cannot be implemented. The temporal association between 4F-PCC administration and bleeding resolution does not establish efficacy, safety, or equivalence to APCC.

## 1. Introduction

Acquired hemophilia A (AHA) is a rare autoimmune bleeding disorder caused by neutralizing autoantibodies against coagulation factor VIII (FVIII). It typically presents with new-onset soft-tissue, mucosal, muscular, or urinary bleeding, no prior bleeding history, and isolated prolongation of activated partial thromboplastin time (APTT) [1,2].

**Figure 1:**
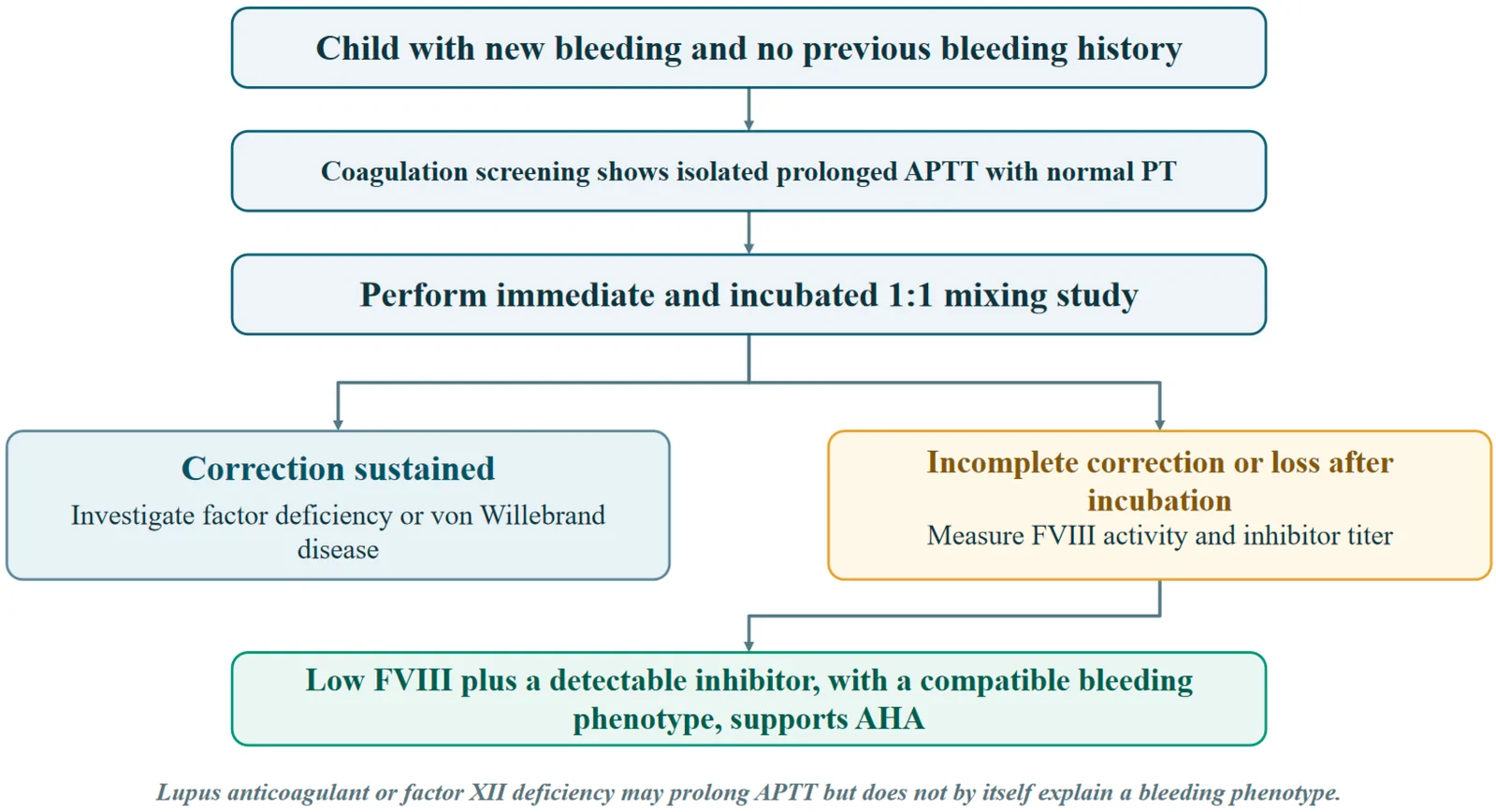
Diagnostic reasoning in suspected pediatric acquired hemophilia A. This simplified diagram summarizes the assessment sequence; it is not a validated clinical decision rule.

AHA occurs predominantly in older adults and postpartum women and is exceptionally uncommon in children [3]. A 20-year French retrospective cohort identified only eight pediatric cases, underscoring the scarcity of child-specific data and the extent to which management is extrapolated from adult evidence [4]. In a child with markedly reduced FVIII activity, the differential diagnosis includes congenital hemophilia A, von Willebrand disease, lupus anticoagulant, and other intrinsic-pathway abnormalities. Mixing studies and inhibitor testing are central to distinguishing an acquired FVIII inhibitor from isolated factor deficiency [1,5,6]. A simplified diagnostic reasoning sequence is shown in Figure 1.

Management has two parallel objectives: control of clinically relevant bleeding and eradication of the FVIII inhibitor. International recommendations identify recombinant activated factor VII (rFVIIa), activated prothrombin complex concentrate (APCC), and recombinant porcine FVIII as hemostatic options [1]. Non-activated four-factor prothrombin complex concentrate (4F-PCC) contains non-activated factors II, VII, IX, and X, whereas APCC contains activated coagulation factors and has bypassing activity. These preparations are pharmacologically distinct and are not interchangeable. Evidence from other indications, including vitamin K antagonist reversal, cannot establish the efficacy of 4F-PCC for AHA. We report diagnostic reasoning, constraint-driven hemostatic decision-making, and long-term follow-up in a child in whom recommended hemostatic agents could not be implemented.

## 2. Case Presentation

### 2.1. Case-Report Design and Source Data

**Figure 2:**
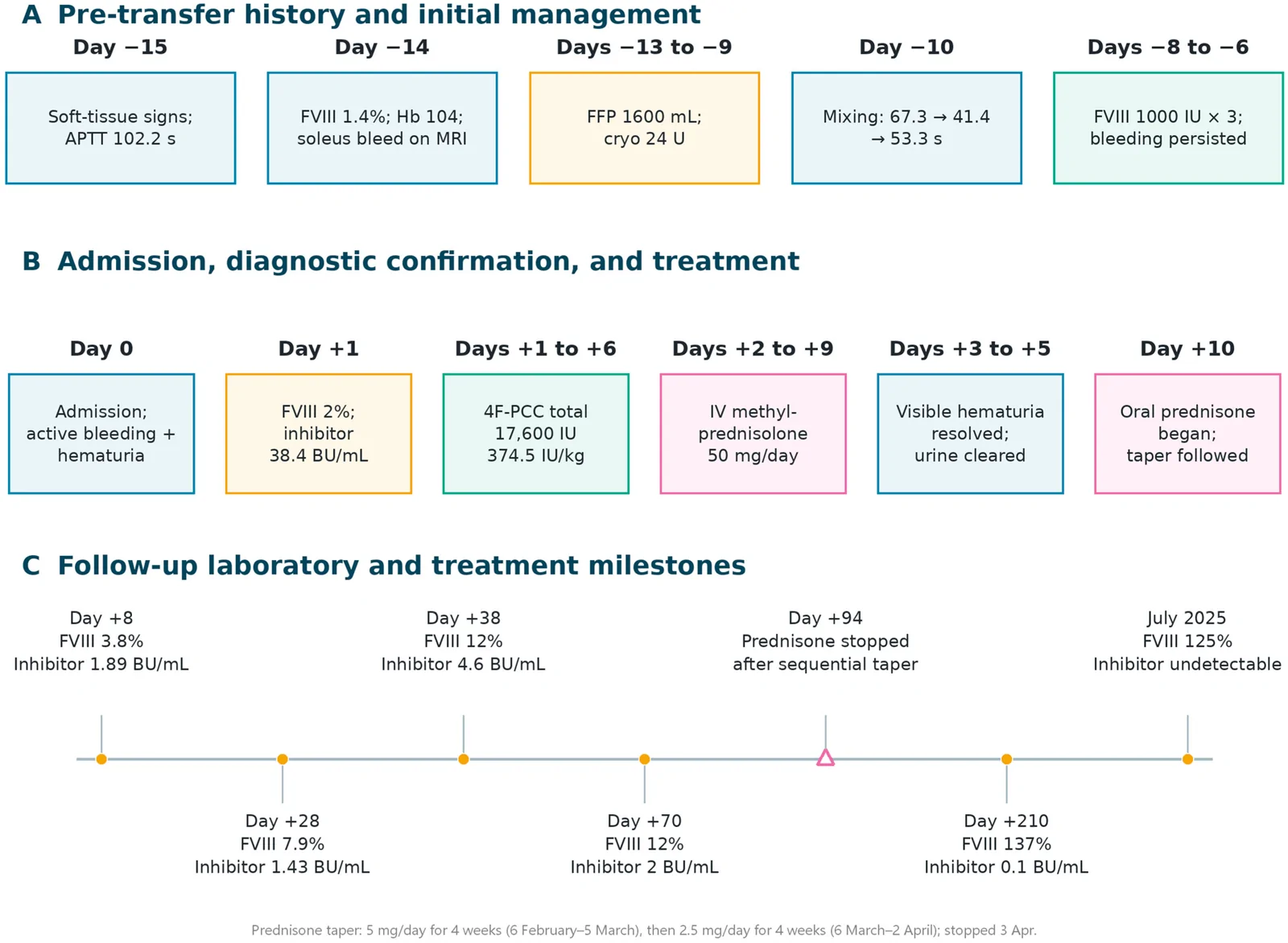
Clinical timeline of diagnosis, individualized hemostatic management, and follow-up. (**A**) Pre-transfer evaluation and replacement therapy. (**B**) Diagnostic confirmation and individualized hemostatic and immunosuppressive treatment after transfer. (**C**) Laboratory recovery and long-term follow-up. Day 0 was hospital admission; subsequent day numbers are relative to admission. Long-term follow-up is shown by month and year when an exact day was not documented. Milestones are sequential and not proportional to elapsed time. Results from different laboratories and assay formats are presented as separate measurements. APTT, activated partial thromboplastin time; BU, Bethesda unit; FFP, fresh frozen plasma; FVIII, factor VIII; 4F-PCC, four-factor prothrombin complex concentrate. The exact date of complete resolution of epistaxis and calf findings was not documented.

This retrospective case report was prepared in accordance with the CARE guideline [7]. The authors reviewed inpatient medical records, original laboratory reports, medication orders and administration records, imaging reports, follow-up records, and de-identified communications with the patient’s family. For clarity, the date of hospital admission was designated Day 0; earlier and later events are reported as days relative to admission where the exact date was documented. 4F-PCC doses were calculated by body weight, whereas corticosteroid doses were calculated by body surface area according to the documented treatment approach. Dose-per-kilogram values are reported only for 4F-PCC and use the admission weight of 47 kg. Results generated by different laboratories were not assumed to be directly comparable. Generative AI tools were used in the preparation of this paper: OpenAI ChatGPT (GPT-5.5 Pro) was used for language editing, and OpenAI Codex (GPT-5.3-Codex) was used to prepare Figure 1 and Figure 2 based on clinical information and figure instructions provided by the authors. The authors reviewed and verified all AI-assisted text and figure content.

### 2.2. Clinical History and Presentation

An 11-year-old previously healthy girl was admitted on Day 0 with left-leg pain and swelling that had been present for more than half a month and recurrent epistaxis for approximately two weeks. She had no previous abnormal bleeding or family history of an inherited bleeding disorder. She had not reached menarche. At admission, she was 147 cm tall and weighed 47 kg; her weight was 48 kg one week later. Before admission, ultrasonography showed calf and upper-arm soft-tissue abnormalities, and magnetic resonance imaging showed edema and bleeding in the left soleus muscle. Hemoglobin was 104 g/L at the referring hospital. At transfer, she had ecchymosis, calf swelling, and gross hematuria.

An inherited FVIII deficiency was initially considered. Before transfer, she received fresh frozen plasma (400 mL on Days −13, −12, −10, and −9; cumulative 1600 mL), cryoprecipitate (8 U daily on Days −13 to −11; cumulative 24 U), and three 1000-IU doses of plasma-derived FVIII between Days −8 and −6. Bleeding remained clinically active, and the APTT remained prolonged (108.5 s on Day −6). Persistent bleeding and APTT prolongation despite plasma products and FVIII replacement increased suspicion of an inhibitor-mediated process.

Specialist testing on Day +1 showed an APTT of 100.2 s, FVIII activity of 2%, uncorrected immediate and incubated mixing tests, and an FVIII inhibitor. The combination of new-onset bleeding without a prior bleeding history, isolated prolonged APTT, low FVIII activity, and inhibitor detection supported AHA. Etiologic evaluation included assessment for infection, recent vaccination or medication exposure, autoimmune disease, malignancy, and other potential secondary causes. No clear associated condition was identified from the available clinical and laboratory evaluation.

### 2.3. Diagnostic Assessment

At the referring hospital (Day −10), the APTT was 67.3 s, the immediate 1:1 mixture was 41.4 s, and the 1:1 mixture after 2 h was 53.3 s. Specialist testing on Day +1 showed a patient APTT of 100.2 s, normal control APTT of 36.3 s, immediate 1:1 mixture of 57.2 s, 1:1 mixture after incubation of 77.2 s, and post-incubation 1:1 mixture of 62.6 s. The specialist laboratory interpreted the immediate and incubated mixing tests as uncorrected, supporting an intrinsic coagulation-factor inhibitor. The incubation conditions and local correction criterion were unavailable.

A Bethesda assay on a sample collected on Day 0 yielded 4.32 BU/mL. A separate sample collected on Day +1 and tested at the coagulation reference laboratory at Chongqing Hemophilia Comprehensive Management Center by a Nijmegen-modified Bethesda assay yielded 38.4 BU/mL. This was the highest documented inhibitor titer in the records reviewed and the principal confirmatory measurement. The values are reported independently because the samples were collected on different days and tested in different laboratories using different assay formats; they should not be interpreted as a 24 h biological trend. The Nijmegen-modified Bethesda result was used as the principal confirmatory value because it was obtained at the specialist hemophilia center using the Nijmegen-modified assay, which improves specificity and reliability for FVIII inhibitor measurement [6]. The sequential coagulation and hematologic findings during diagnosis and follow-up are summarized in Table 1.

### 2.4. Constraint-Driven Hemostatic Management

Bleeding remained clinically active after transfer, with documented muscle hematoma, gross hematuria, and epistaxis. Hemoglobin was 104 g/L and did not subsequently decline; no red-cell transfusion was given. Hemostatic treatment was initiated because clinically relevant bleeding was ongoing. rFVIIa was financially inaccessible to the family because it was not reimbursable under the patient’s applicable medical insurance coverage and the out-of-pocket treatment cost was beyond the family’s financial capacity. APCC was not marketed in mainland China at the time of treatment. Emicizumab was not considered during this episode; at that time, a 30 mg vial reportedly cost RMB 8100 in China, which was beyond the family’s financial capacity. After multidisciplinary discussion with the family about active bleeding, available treatment options, access constraints, and potential thrombotic risk, non-activated 4F-PCC was selected and administered off-label. This report does not propose 4F-PCC as an established AHA treatment.

Human prothrombin complex concentrate (Hualan Biological Engineering Inc., Xinxiang, China) was used. It was a non-activated four-factor preparation containing factors II, VII, IX, and X and heparin; it was not APCC. Treatment began on Day +1. The first six doses were 2000 IU each (42.6 IU/kg per dose) at 8 h intervals. This was followed by three 1200-IU doses (25.5 IU/kg per dose) at intervals of 10, 12, and 14 h, then two 1000-IU doses (21.3 IU/kg per dose) at intervals of 24 and 20 h. Eleven doses totaled 17,600 IU (approximately 374.5 IU/kg). Dose selection, interval extension, and discontinuation were empirical; no validated 4F-PCC regimen exists for AHA. The administration sequence is detailed in Supplementary Table S1.

Gross hematuria gradually lightened after treatment began and was no longer visible by Day +3. Urinalysis continued to show 5 red blood cells/μL; repeat testing on Day +5 showed no urinary red blood cells. Calf swelling and tenderness also persisted after the gross hematuria resolved. These residual findings were the documented reasons for continuing empirical tapering through Day +6. No new major bleeding or clinically apparent thrombosis was documented after treatment; however, systematic surveillance for asymptomatic thrombosis was not performed.

### 2.5. Inhibitor Eradication and Follow-Up

Methylprednisolone 50 mg intravenously once daily was given from Day +2 through Day +9. Oral prednisone began at 20 mg/day on Day +10, was reduced to 10 mg/day from Day +24 through Day +37, 5 mg/day from Day +38 through Day +65, and 2.5 mg/day from Day +66 through Day +93, and was discontinued on Day +94. Corticosteroid doses were calculated according to body surface area; the absolute doses and taper are reported as documented in the medication record. Rituximab and cyclophosphamide were discussed as contingency or escalation options but were not used because of financial and toxicity concerns, remote residence, and the feasibility of oral treatment. Escalation was planned if laboratory response was inadequate or bleeding recurred.

The family reported no missed corticosteroid doses during the taper. Mild gastrointestinal discomfort occurred, but no clinically significant treatment-related adverse effects were reported.

FVIII activity was 12%, and the inhibitor was 4.6 BU/mL on Day +38. On Day +210, FVIII activity was 137%, and the inhibitor was 0.1 BU/mL. At long-term follow-up in July 2025, FVIII activity was 125%, the inhibitor was undetectable, immunosuppression had been withdrawn, and no recurrent major AHA-related bleeding had been reported.

### 2.6. Patient and Family Perspective

During follow-up, the legal guardian reported that the child had experienced no further bleeding and was doing well. The family considered the treatment course successful because the bleeding problem had resolved and the child subsequently remained clinically stable.

## 3. Discussion

### 3.1. Diagnostic Challenges in Pediatric AHA

Pediatric AHA is diagnostically challenging because its rarity encourages consideration of inherited bleeding disorders and because APTT abnormalities may have more than one explanation. In this patient, the new bleeding phenotype, isolated marked APTT prolongation, low FVIII activity, inhibitor-type mixing pattern, persistent bleeding and APTT prolongation despite replacement therapy, and inhibitor detection supported AHA [1,3,4,5,6]. Reduced FXII activity was identified during the initial evaluation; although it can prolong APTT, it does not explain the bleeding phenotype. No secondary condition was identified during etiologic evaluation; however, an unrecognized association cannot be excluded completely in a retrospective single-case report.

### 3.2. Hemostatic Management When Recommended Agents Are Inaccessible

The clinically important feature of this case was the inability to implement recommended hemostatic agents. rFVIIa was financially inaccessible to the family because it was not reimbursed under the applicable medical insurance coverage and the out-of-pocket cost exceeded the family’s financial capacity; APCC was not marketed in mainland China at the time of treatment. Recommended options for clinically relevant AHA bleeding include rFVIIa, APCC, and recombinant porcine FVIII [1,8]. Non-activated 4F-PCC contains non-activated factors II, VII, IX, and X, including factor VII, whereas APCC contains activated coagulation factors and has bypassing activity. The preparations are pharmacologically distinct. Evidence concerning 4F-PCC in vitamin K antagonist reversal does not establish its efficacy for AHA, and comparative superiority for AHA cannot be inferred from this single case or from evidence in other indications.

The 4F-PCC decision was made after multidisciplinary discussion with the family, considering ongoing bleeding, treatment availability, affordability, and uncertainty about thrombotic risk. It should be understood as an individualized off-label decision under the circumstances of this case, not as a recommended alternative or protocol. In settings where guideline-recommended agents are available, 4F-PCC should not be regarded as an equivalent substitute.

### 3.3. Clinical Response, Safety, and Causal Uncertainty

Gross hematuria resolved after 4F-PCC was initiated, whereas microscopic hematuria and calf findings resolved more slowly. This establishes timing only. Previous fresh frozen plasma, cryoprecipitate, and plasma-derived FVIII; methylprednisolone initiated approximately 24 h after 4F-PCC; the natural evolution of bleeding; possible spontaneous improvement; and the uncontrolled single-case design preclude causal attribution. Because methylprednisolone was initiated within approximately 24 h of the first 4F-PCC dose and gross hematuria resolved approximately 36–48 h after 4F-PCC initiation, the temporal overlap further precludes attribution of hemostatic improvement to either intervention alone. The observed sequence does not establish efficacy, safety, or equivalence of 4F-PCC for AHA.

Thromboembolism is a concern with 4F-PCC, although much of the available evidence concerns anticoagulant reversal in adults [9]. No clinically documented thrombosis occurred, and D-dimer was mildly elevated during active bleeding and subsequently normalized according to the available records. However, systematic laboratory and vascular imaging surveillance for asymptomatic thrombosis was not performed. The absence of documented thrombosis should not be interpreted as proof of safety.

### 3.4. Inhibitor Eradication and Long-Term Outcome

Bleeding control and inhibitor eradication are parallel treatment goals. Adult recommendations and registry data support risk-adapted immunosuppression, but pediatric evidence remains sparse [1,3,10]. The recent French pediatric cohort of eight cases likewise illustrates the rarity of AHA in children and the historical reliance of pediatric care on adult standards [4]. In this case, the specialized inhibitor titer was 38.4 BU/mL while FVIII activity was approximately 2%. Adult recommendations generally favor risk-adapted immunosuppression, with more intensive regimens considered in patients with higher inhibitor titers or very low FVIII activity; however, pediatric evidence remains limited. The treatment strategy reflected individualized assessment of toxicity, feasibility, treatment access, and planned escalation if response was inadequate [1]. Corticosteroid monotherapy was selected after discussion of feasibility and family circumstances, with rituximab and cyclophosphamide retained as escalation options rather than considered unnecessary. This course should not be interpreted as evidence that corticosteroid monotherapy is preferable for pediatric patients with similar inhibitor titers. Sustained normal FVIII activity, an undetectable inhibitor, withdrawal of immunosuppression, and no relapse through July 2025 are important strengths of the report. Continued FVIII monitoring remains appropriate because FVIII activity is more sensitive than APTT for detecting recurrence [1,8].

This case also illustrates the economic and health-system consequences of treatment access constraints. The family could not afford rFVIIa, while APCC was unavailable locally, so the treatment plan was shaped by both clinical need and the family’s financial circumstances. Economic models in inherited hemophilia have emphasized that treatment costs, health-related quality of life, and utility assumptions are relevant to evaluating care [11]; those findings cannot be directly extrapolated to pediatric AHA, but they support the need to consider affordability and access when describing real-world treatment decisions.

### 3.5. Limitations

This report is limited by its retrospective, uncontrolled single-patient design; concomitant treatments; absence of a validated AHA-specific bleeding endpoint; incomplete documentation of the resolution dates for epistaxis and calf findings; interlaboratory and assay-format differences; incomplete details of sampling times and some laboratory methods; and no systematic surveillance for asymptomatic thrombosis. Because this is one case with a favorable outcome, it cannot show whether the same approach would be effective or safe in children with different bleeding severity, inhibitor characteristics, comorbidities, or access to recommended agents.

## 4. Conclusions

Pediatric AHA is diagnostically and therapeutically challenging. This case highlights the importance of early inhibitor recognition and transparent, multidisciplinary decision-making when standard treatment access is constrained. Management requires integration of clinical severity, laboratory evidence, treatment accessibility, family circumstances, and safety uncertainty. In this patient, bleeding resolution occurred temporally during off-label non-activated 4F-PCC administration; this observation does not establish efficacy or safety. Pediatric-specific evidence for diagnosis, bleeding management, inhibitor eradication, and long-term monitoring is still needed.

## Figures and Tables

**Table 1:** Sequential coagulation and hematologic parameters during diagnosis and follow-up.

Relative Time Point	APTT (s; RI 25.1–36.5)	FVIII Activity (%; RI 50–150)	FVIII Inhibitor (BU/mL; RI 0–0.6)	Clinical Context; Reference Intervals: PT 9.9–12.8 s; TT 10.3–16.6 s; Fibrinogen 238–498 mg/dL (2.38–4.98 g/L)
Day −15, county hospital	102.2	Not tested	Not tested	PT 10.70 s; TT 12 s; fibrinogen 4.28 g/L; D-dimer 0.79 mg/L; FDP 6.24 mg/L; calf and upper-arm soft-tissue abnormalities on ultrasonography.
Day −14, outside hospital	NA	1.4%	Not tested	Hemoglobin 104 g/L; FVIII method not recorded; FXII 27.7%; vWF activity 54.0%; left soleus edema and bleeding on MRI.
Day −10, outside hospital	67.3	NA	Not tested	Immediate 1:1 mixing result 41.4 s; 2 h mixture 53.3 s.
Days −8 to −6, outside hospital	71.8–108.5	1.7%	Not tested	Three plasma-derived FVIII doses of 1000 IU were given; bleeding and APTT abnormality persisted.
Day 0 sample	NA	NA	4.32	Wuhan Kindstar Medical Laboratory Co., Ltd.; Bethesda assay; report date unavailable; heat inactivation unknown.
Day +1 sample	100.2	2%	38.4	Chongqing Hemophilia Comprehensive Management Center; Nijmegen-modified Bethesda assay; immediate and incubated mixing tests uncorrected.
Day +8	68.8	3.8%	1.89	Mild residual left-calf swelling; no new major bleeding.
Day +28	53.8	7.9%	1.43	Outpatient follow-up; laboratory and assay method not recorded.
Day +38	Not tested	12%	4.6	Local-hospital sample sent to an external laboratory; assay details unavailable.
Day +70	49.6	12%	2	Follow-up; laboratory and assay method not recorded.
Day +210	Not tested	137%	0.1	Laboratory remission milestone; prednisone was stopped on Day +94.
July 2025 follow-up	NA	125%	0	No immunosuppression; no recurrent major AHA-related bleeding reported.

Abbreviations: AHA, acquired hemophilia A; APTT, activated partial thromboplastin time; BU, Bethesda unit; FVIII, factor VIII; NA, value not available in the records reviewed. Day 0 was the date of hospital admission. Results from different laboratories or assay formats are not necessarily directly comparable. For some measurements, the interval between sample collection and preceding treatment was unavailable. Reference intervals are displayed for tests with available laboratory ranges; reference intervals for D-dimer and FDP were not available in the reports reviewed.

## Data Availability

The de-identified clinical data supporting the findings of this case report are contained within the article and its Supplementary Materials. Additional de-identified information may be available from the corresponding author on reasonable request and subject to appropriate institutional approval.

## Supplementary Materials

The following supporting information can be downloaded at: https://www.mdpi.com/article/10.3390/jcm15207859/s1; Table S1: Detailed 4F-PCC administration record (relative day and time, dose, dose per kilogram, and interval from the previous dose); Table S2: CARE Checklist for Case Reports.
