# Supplementary material for: Managing Pediatric Acquired Hemophilia A When Guideline-Recommended Bypassing Agents Are Unavailable: A Case Report
Source: J Clin Med. 2026 Oct 12;15(20):7859. doi: 10.3390/jcm15207859 (PMC13706168; doi:10.3390/jcm15207859)
Supplement: Supplementary file 1 [file jcm-15-07859-s001.zip › jcm-4565618-supplementary/jcm-4565618-supplementary.pdf]

Supplementary Table S1. Detailed 4F-PCC Administration Record

Non-activated four-factor prothrombin complex concentrate (4F-PCC); admission was on Day 0. Dose-per-kilogram values are calculated using the admission weight of 47 kg.

Dose no.	Relative day and time	Dose (IU)	Dose (IU/kg)	Interval from previous dose
1	Day +1, 12:00	2000	42.6	—
2	Day +1, 20:00	2000	42.6	8 h
3	Day +2, 04:00	2000	42.6	8 h
4	Day +2, 12:00	2000	42.6	8 h
5	Day +2, 20:00	2000	42.6	8 h
6	Day +3, 04:00	2000	42.6	8 h
7	Day +3, 14:00	1200	25.5	10 h
8	Day +4, 02:00	1200	25.5	12 h
9	Day +4, 16:00	1200	25.5	14 h
10	Day +5, 16:00	1000	21.3	24 h
11	Day +6, 12:00	1000	21.3	20 h

Total administered: 17,600 IU (approximately 374.5 IU/kg). The six 2000-IU doses were administered at 8 h intervals, followed by three 1200-IU doses at 10, 12, and 14 h intervals and two 1000-IU doses at 24 and 20 h intervals. Gross hematuria was no longer visible by Day +3; urinalysis showed 5 red blood cells/μL before no urinary red blood cells were detected on Day +5. Calf swelling and tenderness persisted after gross hematuria resolved and were documented reasons for continuing the taper through Day +6. These temporal observations do not establish a causal effect of 4F-PCC.
