# Supplementary material for: Managing Pediatric Acquired Hemophilia A When Guideline-Recommended Bypassing Agents Are Unavailable: A Case Report
Source: J Clin Med. 2026 Oct 12;15(20):7859. doi: 10.3390/jcm15207859 (PMC13706168; doi:10.3390/jcm15207859)
Supplement: Supplementary file 1 [file jcm-15-07859-s001.zip › jcm-4565618-supplementary/JCM_CARE_Checklist.pdf]

CARE Checklist for Case Reports

Managing Pediatric Acquired Hemophilia A When Guideline-Recommended Bypassing Agents Are Unavailable: A Case Report

Item	Topic	CARE requirement	Manuscript location
1	Title	The diagnosis or intervention of primary focus followed by "case report".	p.1, Title
2	Keywords	2–5 keywords that identify diagnoses/interventions, including "case report".	p.1, Keywords (final: acquired hemophilia A; child; factor VIII inhibitor; hemostatic management; case report)
3a	Abstract—Introduction	What is unique about this case and what it adds to the literature.	p.1, Abstract—Background/Objectives
3b	Abstract—Symptoms/findings	Main symptoms and important clinical findings.	p.1, Abstract—Case
3c	Abstract—Diagnosis/intervention/outcome	Main diagnoses, therapeutic interventions, and outcomes.	p.1, Abstract—Case/Results
3d	Abstract—Conclusion	Main take-away lesson.	p.1, Abstract—Conclusions
4	Introduction	Brief background and why the case is unique or clinically relevant.	pp.1–2, Section 1
5a	Patient information—Demographics	De-identified demographic information.	p.3, Section 2.3
5b	Patient information—Main concerns	Patient's main concerns and symptoms.	p.3, Section 2.3
5c	Patient information—History	Medical, family, and relevant psychosocial history.	pp.3,5, Sections 2.3–2.5
5d	Patient information—Past interventions	Relevant past interventions and outcomes.	p.3, Section 2.3
6	Clinical findings	Relevant physical examination and clinical findings.	p.3, Section 2.3
7	Timeline	Important dates and events in chronological order.	p.6, Figure 1
8a	Diagnostic assessment—Methods	Diagnostic methods including laboratory and imaging assessment.	pp.2–4, Sections 2.2–2.3; Table 1
8b	Diagnostic assessment—Challenges	Diagnostic challenges and uncertainties.	pp.2–3, Sections 2.2–2.3; p.7, Section 3.1
8c	Diagnostic assessment—Diagnosis/differential	Diagnosis and differential diagnoses considered.	pp.2–3, Sections 1 and 2.3
8d	Diagnostic assessment—Prognosis	Prognostic characteristics where applicable.	Not applicable
9a	Therapeutic intervention—Types	Types of therapeutic interventions.	pp.3–5, Sections 2.3–2.5
9b	Therapeutic intervention—Administration	Dosage, strength, duration, and administration details.	p.5, Sections 2.4–2.5; Supplementary Table S1
9c	Therapeutic intervention—Changes	Changes in intervention and rationale.	p.5, Sections 2.4–2.5
10a	Follow-up and outcomes—Clinician/patient outcomes	Clinician- and patient/family-assessed outcomes.	Section 2.5; Patient and Family Perspective
10b	Follow-up and outcomes—Tests	Important follow-up diagnostic or laboratory results.	Table 1; Figure 1; Section 2.5
10c	Follow-up and outcomes—Adherence/tolerability	Intervention adherence and tolerability.	Section 2.5 (final manuscript: no missed corticosteroid doses; mild gastrointestinal discomfort; no clinically significant treatment-related adverse effects)
10d	Follow-up and outcomes—Adverse events	Adverse and unanticipated events.	Sections 2.4 and 3.3
11a	Discussion—Strengths/limitations	Strengths and limitations of the case.	pp.7–8, Sections 3.1–3.5
11b	Discussion—Literature	Relevant medical literature.	pp.7–8, Sections 3.1–3.4
11c	Discussion—Rationale	Scientific rationale for conclusions and interpretations.	pp.7–8, Sections 3.2–3.4
11d	Discussion—Take-away lesson	Main take-away lesson in a concluding paragraph.	p.8, Section 4
12	Patient perspective	Patient or family perspective on the treatment experience.	Patient and Family Perspective section before Discussion
13	Informed consent	Written informed consent for publication.	Institutional statements section—Informed Consent Statement
